# Lessons Learned in Improving the Quality of a Free Reproductive Health Hotline in Benin

**DOI:** 10.9745/GHSP-D-22-00296

**Published:** 2023-12-22

**Authors:** Cyprien Zinsou, Ghyslain Guedegbe, Leonce Dossou, Judith Ognin, Ando Tiana Raobelison, Lola Flomen, Paul Bouanchaud

**Affiliations:** a Association Béninoise pour le Marketing Social et la Communication pour la Santé, Cotonou, Benin.; b Independent consultant, Montreal, Canada.; c Population Services International, Washington, DC, USA.

## Abstract

**Introduction:**

Since 2006, the Ligne Verte project—a toll-free national hotline that provides counseling and provider linkages to family planning, HIV, and sexually transmitted infection services—has been implemented by the Association Béninoise pour le Marketing Social et la Communication pour la Santé (ABMS) in Benin. Annual evaluations have been conducted to monitor client satisfaction, with feedback mechanisms to learn from callers and staff, aiming to continually improve the program. We document lessons learned from these evaluations, outline the adaptation process to improve the quality of the hotline, present results from Ligne Verte caller and counselor studies, and describe how the studies fed client-driven changes to the project.

**Methods:**

Annual rounds of semistructured phone interviews with Ligne Verte callers and call center advisors were conducted. The sample of clients interviewed included consenting men and women aged 18 years and older who had used Ligne Verte services in the 3 months preceding the evaluation. All advisors working on the helpline were also eligible for inclusion in qualitative interviews.

**Results:**

From 2013 to 2020, most callers were satisfied with the Ligne Verte project. Users reported appreciating the accurate, timely, and confidential support they received from the helpline but noted the need to expand the service to other local languages and improve call quality. Call center advisors requested mental health support. In employing a systematic approach to program improvement, ABMS was able to add extended language services, upgrade their telecommunication hardware, and provide a psychologist for call center advisors. These program adaptations have increased caller satisfaction by 20 percentage points, and in 2020, 95% of callers reported being satisfied with their Ligne Verte experience.

**Conclusion:**

Taking a consumer-centered approach, listening to user feedback, and making evidence-based recommendations to improve the Ligne Verte project has led to an overall increase in caller satisfaction.

## Abstract (precis)

Benin's Ligne Verte reproductive health hotline is filling information gaps and linking callers to services while getting regular feedback from its users to improve the service provided.

## Teaser

**Key Findings:**

- In 2006, the Ligne Verte phone hotline was created to provide individuals in Benin with free access to information about sexual and reproductive health topics at a time when unmet need for contraception was high and young women and adolescent girls were not being reached with reproductive health services.
- Annual satisfaction surveys are conducted to improve the hotline to better address caller's needs and support call counselors.
- The collaborative improvement process allowed the research team to develop a clearer understanding of the program's needs and weaknesses and thus make more realistic, affordable, actionable, and evidence-based recommendations for improvements.

**Key Implication:**

- To generate and maintain high client satisfaction, health program managers designing similar programs to Ligne Verte should prioritize end-user perspectives, support staff morale, and create continuous feedback loops for program improvement.

## INTRODUCTION

Benin's high maternal mortality rate of 397 per 100,000 live births suggests that many women living in Benin could benefit from on-demand, high-quality reproductive health information and services.^1^ In 2006, unmet need for contraception was around 28% in Benin,^1^ with low reported uptake of modern methods (6%).^2^ Across the country, young women and adolescent girls were not being reached with the reproductive health services they needed to prevent unwanted pregnancies. Knowledge of HIV and sexually transmitted infections was low.^2^ In Benin, sexually active individuals had limited opportunities to access personalized and credible reproductive health information in real time.

To provide accurate and accessible reproductive health information, in 2006, the Association Béninoise pour le Marketing Social et la Communication pour la Santé (ABMS) initiated the Ligne Verte project: a toll-free national hotline that provides counseling and linkages to family planning (FP), HIV, and sexually transmitted infection services. At that time, the key conditions for establishing the hotline included increasing mobile phone penetration, expanding mobile network coverage, and shifts in government health priorities.

The growth of mobile phone ownership and network coverage in Benin has created an opportunity for reproductive health programs to invest in phone-based social behavior change campaigns. In the most recent Demographic and Health Survey in 2017/18, 84% of people in Benin owned a mobile phone.^1^ Evidence from Burkina Faso, Tanzania, and India suggests that when adjusting for sociodemographic characteristics, women who own mobile phones are significantly more likely to adopt modern contraception methods, use a skilled birth attendant, and take up postnatal care.^3^^–^^5^ With national 3G mobile network coverage of 89%, Benin has one of the fastest-growing information, technology, and communication sectors in West Africa.^6^ Given Benin's high mobile phone and network penetration, health promotion organizations like ABMS pivoted to phone lines to deliver consumer-centered reproductive health information. Furthermore, Benin's Ministry of Health is prioritizing the implementation of digital health platforms that deliver reliable, affordable, and accessible health information. One of the goals of the latest National Digital Health Strategy (2018–2022) is to use digital tools to reduce maternal and infant mortality.^7^

The growth of mobile phone ownership and network coverage in Benin has created an opportunity for reproductive health programs to invest in phone-based social behavior change campaigns.

Having been operational for more than 17 years, the Ligne Verte project has been present through this period of growth in mobile phone use and shifts in Ministry of Health policy. The project has sought to improve the hotline to better address callers' health information and service needs. Key to this process is Ligne Verte's annual caller satisfaction survey, the results of which feed into program adaptations. In this article, we document lessons learned from the annual evaluations, outline the adaptation process to improve the quality of the hotline, present results from Ligne Verte caller and counselor studies, and describe how the studies fed client-driven changes to the project. We describe implications for reproductive health program management and include recommendations for implementers who desire to adapt the Ligne Verte approach for other health promotion programs.

## LIGNE VERTE PROJECT

In 2006, Ligne Verte was created by ABMS, a Beninese nongovernmental organization and member of the Population Services International network. ABMS' mission is to contribute effectively and sustainably to the improvement of the health conditions of the Beninese population, particularly its most vulnerable communities, through social marketing and communication in collaboration with the government, development partners, and the private sector.

ABMS created Ligne Verte to provide the whole population of Benin, particularly adolescents and young adults, with access to information about sexual and reproductive health (SRH) and to allow them to talk openly, free of judgment, and for free about their concerns. By dialing 7344 from anywhere in the country, callers can speak with trained call center counselors to obtain accurate and credible information about FP, the prevention of sexually transmitted infections and unwanted pregnancies, and other health information that has been added to the service over time. Each caller has the option of being connected with a brick-and-mortar clinic. The specific referral depends on their health needs but might be to an ABMS-franchised ProFam (FP and reproductive health) clinic, a *centre jeunes amour et vie* (youth-friendly SRH services and rights center), a public sector health center, or other nearby clinic run by a nongovernmental organization.

The call center is staffed with phone counselors who must have at least 2 years of post-high school study, some prior experience in working in a call center, and be able to speak French and several Beninese languages. Counselors receive a comprehensive in-person training package on-site that includes call-answering techniques, communication skills, and substantive content around ABMS's key health areas and themes. The training is participatory in nature, with 2 full days used for role-playing call scenarios.

Ligne Verte is free and accessible in all 12 geographical *departements* of Benin, Monday to Friday from 9 a.m. to 9 p.m. and on Saturdays from 9 a.m. to 5 p.m. The service receives approximately 350 calls per day. Each counselor has a daily goal of completing 150 calls while focusing on the satisfaction of their callers, a key goal of the program. The service is promoted through radio and television advertisements for ABMS products or services in which the Ligne Verte number is provided for more information, directly through flyers and posters in centres jeunes amour et vie, and by word of mouth from health providers. In addition, ABMS has recently introduced a chatbot, “Tata Annie,” to provide FP and menstrual health and hygiene information for young women and men in Benin. Although full integration between the helpline and chatbot is not possible with current funding, the chatbot provides the Ligne Verte number to users looking for more information.

## METHODS

To evaluate caller satisfaction, ABMS researchers conducted annual phone interviews from 2013 to 2020 with a random sample of Ligne Verte callers. A structured questionnaire was used to guide the interview and focused on reasons for satisfaction or dissatisfaction with Ligne Verte's service quality. Starting in 2018, the evaluation design expanded to include qualitative interviews with the call center advisors, focusing on the challenges of their work and their interactions with callers.

### Sample Population

The target population for the Ligne Verte satisfaction evaluations was men and women aged 18 years and older who had used Ligne Verte services in the 3 months preceding the survey, as well as current (since 2018) call center advisors. Callers were randomly selected using their numbers recorded in a database of callers of the reference period. To ensure geographical representation, we stratified the caller sample geographically to cover all 12 departements in the country. All potential respondents gave their voluntary informed consent to participate. No personally identifying information was collected during the short (approximately 10 minutes) telephone survey. Each year since 2018, all 4–6 call center advisors have given their informed consent to be interviewed and have been included in the qualitative study. The sample size of callers for each wave of data collection can be seen in Table 1.

**TABLE 1.** Ligne Verte National Reproductve Health Hotline Quality Assessment Sample Sizes, 2013 to 2020, Benin

Year	Callers Sampled, No.
2013	254
2014	925
2015	1,036
2016	1,246
2017	1,252
2018	1,296
2019	1,212
2020	1,272

### Data Collection

For callers, the questionnaire centered on some basic sociodemographic characteristics, reasons for calling, satisfaction with various elements of the service, including the call advisors' ability to listen and deliver timely solutions, and overall experience (Supplement). For call center advisors, the interviews focused on their experience working on Ligne Verte, challenges related to service delivery, and potential solutions to resolve these difficulties. Data were collected from callers using phone interviews and were recorded electronically via smartphones using the KoboCollect app. Data quality was monitored by the ABMS and Population Services International research teams. Data from the interviews with callers were analyzed using Stata 15. Associations between different variables were tested at the 5% significance level. Open response content collected from callers was analyzed to explore the main reasons for satisfaction and dissatisfaction. A content analysis of call center advisor responses was conducted to examine their perception of work, document challenges, and potential solutions.

### Ethical Approval

All client satisfaction data presented here consist of routine program monitoring data. A nonresearch determination was given to the secondary analysis presented in this article through the Population Services International Research Ethics Board determination process.

## RESULTS

### Sociodemographic Characteristics of Callers

Across all years of data collection, the average age of callers interviewed was between 28 and 29 years, and men have tended to outnumber women in the sample. From 2016 to 2020, women accounted for 38%, 38%, 40%, 31%, and 42% of the callers surveyed, respectively (Table 2 shows the background demographics for the 2020 study). Overall, callers were more likely to be male, live in the south of the country, and work in a professional role when compared to the general population.^1^

**TABLE 2.** Sociodemographic Characteristics of Sample of Ligne Verte Hotline Callers in 2020, Benin

	No. (%)
Geographic area	
Atacora-Donga	105 (8.3)
Borgou-Alibori	78 (6.1)
Atlantic-Littoral	500 (39.3)
Zou-Collines	207 (16.3)
Mono-Couffo	152 (11.9)
Ouémé-Plateau	230 (18.1)
Sex	
Male	734 (57.7)
Female	538 (42.3)
Age group, years	
18–24	317 (24.9)
25–34	351 (27.6)
35 and older	170 (13.4)
Missing	434 (34.1)
Profession	
Learners (students, apprentices)	347 (27.3)
Craftsmen	249 (19.6)
Housewives	58 (4.6)
Traders	181 (14.2)
Teachers and other employees	277 (21.8)
Farmers	53 (4.2)
Self-employed and other professions	60 (4.7)
Total	1,272 (100.0)

### Caller Topics

According to the respondents sampled in each evaluation, the main caller topics for Ligne Verte from 2017 were FP and other reproductive health information, HIV/AIDS, malaria, and diarrheal diseases. Although FP and SRH remained the most reported reasons for calling, starting in 2018, many more callers started inquiring about sanitation (Table 3). In addition, in 2020, 6.1% of callers wanted COVID-19 prevention information and data on the spread of the disease across Benin, and the counselors were trained to provide information based on the Benin government guidance. Those asking about symptoms were referred to the national COVID-19 government helpline.

**TABLE 3.** Ligne Verte Hotline Caller Topics From 2017 to 2020, Benin

Topic (Description)	2017, % N=1,252	2018, % N=1,296	2019, % N=1,212	2020, % N=1,272
Family planning (information about methods and products for family planning that are available in Benin)	35.5	33.4	29.8	47.2
Other reproductive health information (including information about places where further support or information may be found relating to family planning, cervical cancer prevention or treatment, etc.)	20.3	30.0	29.7	16.3
HIV (information about prevention and treatment of HIV)	16.9	6.3	4.2	7.5
Malaria (information about prevention and treatment of malaria)	5.7	2.9	1.5	3.7
Diarrheal diseases (information about causes, prevention, and treatment of diarrheal disease)	2.6	1.3	–	–
Youth magazines and clubs (callers with questions about themes that were included in the Amour et Vie magazines for youth, information about how to create an Amour et Vie club in schools)	4.2	7.9	–	–
Water and sanitation (information about installing ABMS-provided toilettes propres for improved sanitation)	2.0	6.9	17.7	12.0
Information on Ligne Verte (callers with questions about why the helpline is not open 24/7; calls to thank Ligne Verte staff)	1.9	0.5	–	–
Violence (callers reporting experience of gender-based violence for onward referrals)	1.7	1.0	0.8	1.2
Ebola/Lassa fever (calling to have more information on prevention and treatment of these viral hemorrhagic fevers)	0.7	2.2	–	–
National health insurance (for questions about the new health insurance program)	0.2	0.2	0.3	–
COVID-19 (information about prevention, testing, and management)	–			6.1
Other diseases (including information on a range of health conditions including: hepatitis, hemorrhoids, cholera)	1.0	3.8	–	–
Other (non-health-related topics, including information about recruitment to ABMS, participation in competitions)	7.3	3.6	16.0	6.0

Abbreviation: ABMS, Association Béninoise pour le Marketing Social et la Communication pour la Santé.

### Implementing Caller and Call Center Advisor Feedback

**FIGURE 1:**
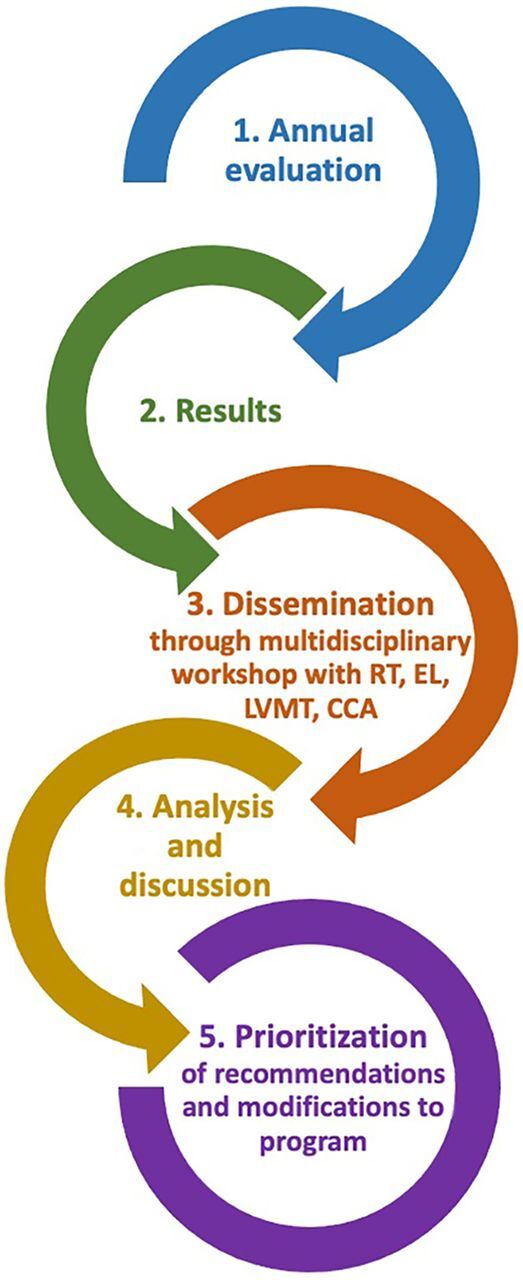
Ligne Verte National Reproductive Health Hotline Improvement Process Abbreviations: CCA, call center advisors; EL, executive leadership; LVMT, Ligne Verte management team; RT, research team.

Each year, the Ligne Verte evaluation feedback and program improvement occurs systematically through a standard process (Figure 1). Immediately after the evaluation is completed, the results are communicated to program managers. ABMS organizes a mini-workshop, bringing together researchers, call center counselors, program managers, and directors to discuss and interpret the results. Actionable improvements are then listed and prioritized, with program changes implemented according to the availability of resources.

In the following section, we present a selection of the challenges that callers and Ligne Verte counselors have identified during the annual evaluations, the process used to make improvements, and an outline of those improvements.

### Language and Communication Challenges and Solution

In a country in which many different languages are spoken, the need to provide support in a wider range of languages was raised by several respondents.

*If the assistants can receive us in different languages (especially Adja) … it would be good because sometimes communication does not go well because of our levels [of French].* —Male, aged 20 years, Aplahoué, 2014

*This service is of great use in addition it is free. But I was never able to speak Bariba. I have to go through my daughter. That's what I dislike the most.* —Female, aged 30 years, Nikki, Borgou, 2017

In 2017, Ligne Verte was operational in French (the official language) and Fon (one of the most widely spoken national languages from the south of the country). Given Benin's huge language diversity, callers requested the response languages be expanded to those spoken more widely in northern Benin.

Upon reviewing the caller and call center advisor feedback, the program intentionally recruited additional call center advisors who could speak a wider range of languages. As of 2020, Ligne Verte was operational in French, Fon, Adja, Goun, Bariba, and Dendi, expanding accessibility to the majority of the country's population.

### Noise and Hardware Challenges and Solution

A few callers noted that there was background noise in the call center, making it difficult to hear the advisor's responses.

*You hear noise there when the assistant picks up the call; in addition, I noticed that the call is not [connected] spontaneously as on the MTN network.* —Male, aged 26 years, Porto-Novo, Ouémé, 2018

In 2018 interviews, several call center advisors also discussed the need to upgrade the workplace setting to ensure that callers had the attention and privacy they needed to discuss reproductive health needs.

*When several advisors receive calls simultaneously, it creates a mess in the room to the point where you can't even hear the caller anymore.* —Call center advisor, aged 33 years, 2018

Call center counselors worked in an open space and answered several calls at a time. Call center advisors often responded to clients on speakerphone, which picked up background conversations.

**FIGURE 2:**
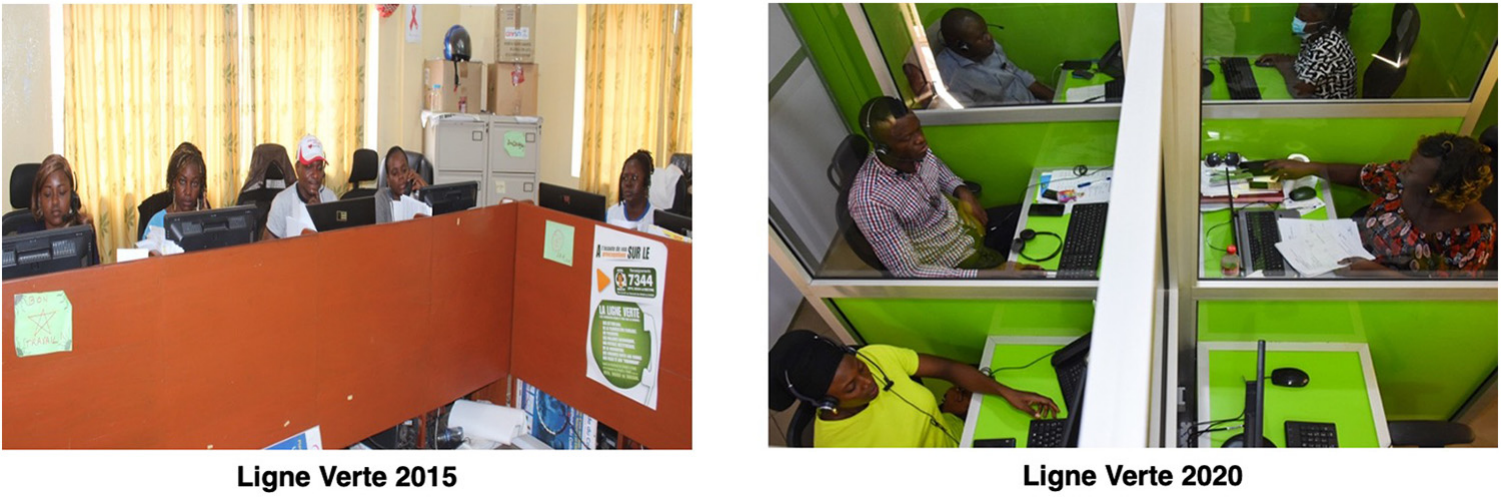
Ligne Verte Hardware Adaptations, From 2015 to 2020, Benin

To resolve background noise issues and ensure the privacy of callers, ABMS invested in creating individual cubicles for each call center advisor and upgraded their telecommunication hardware (inverters, computers, and headphones). Each call center advisor now has a headset to ensure that each caller receives timely, uninterrupted, confidential support (Figure 2).

To resolve background noise issues and ensure caller privacy, ABMS invested in creating individual cubicles for each call center advisor and upgraded their telecommunication hardware.

### Call Center Advisor Perceptions and Morale Solution

Although most call center advisors interviewed said they found their work at Ligne Verte fulfilling, a few noted that a minority of callers treat them disrespectfully.

*The days are never alike at the Ligne Verte. There are days when there are callers who are quite serious in their questions and concerns … But there are days when we receive enough jokers who annoy you and insult you.* —Call center advisor, aged 27 years, 2018

Call center advisors explained that certain callers' frequent use of inappropriate language, threats, and prank calls created stress and discomfort in their workplace.

To ensure that each advisor received emotional support, the program instituted 2 new initiatives: on-site psychological support for call center advisors and year-long employment contracts. Recruiting a psychologist allowed each call center advisor to receive the mental health support they needed to continue to provide quality reproductive health services to their clients. By limiting the work contract to a year with the option of renewal, call center advisors have an annual opportunity to share their concerns before renewing their contract and the project has better oversight into the well-being of their call center advisors. The 1-year contract also allows the project flexibility in ensuring the helpline's needs are being met, for example, through having the flexibility to recruit new staff able to speak a wider range of Beninese languages. Shorter-term contracts (less than 1 year) were considered inappropriate, as opportunities to learn from and implement recommendations from the annual evaluations among the counselors would be limited.

### Trends in Caller Satisfaction

**FIGURE 3:**
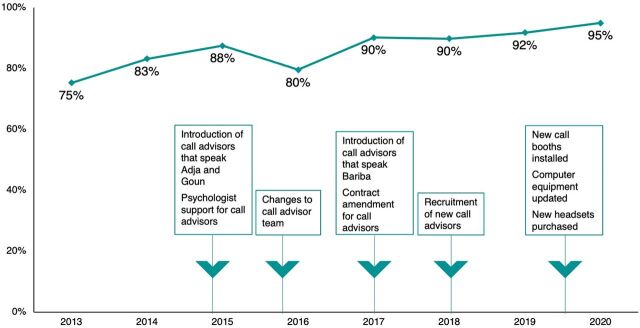
Trends in Ligne Verte Hotline Caller Satisfaction and Concomitant Program Improvements

Since starting annual evaluations in 2013, caller satisfaction has steadily improved overall and in most years (Figure 3), from 74.9% of callers in 2013 to 95% in 2020.

*My wife and I have adopted an FP method and whenever there is a small problem, I call the line and each time I am satisfied.* —Male, aged 27 years, Djougou, Donga, 2020

Callers appreciated the call center advisors' availability, friendliness, and ability to be knowledgeable on a range of FP and HIV prevention services.

*The line is very important in a context where sexuality remains a taboo subject.* —Male, aged 21 years, Natitingou, 2016

In Benin, Ligne Verte played an important role in helping callers feel safe and comfortable to confidentially discuss questions and receive referrals to SRH services.

*Really this number helped me a lot to deal with the problems of my daughter who got pregnant. I even want to call them to thank them.* —Female, aged 34 years, Alibori, 2017

Across Benin, the Ligne Verte project aims to provide parents with the appropriate and relevant knowledge to discuss SRH topics with their children and referrals to other services where appropriate.

## DISCUSSION

The improvement process, especially the annual feedback loops between the call center managers and researchers, has allowed Ligne Verte to better meet the needs of its callers. From 2013 to 2020, satisfaction levels with Ligne Verte have steadily improved. In 2016 we noted a drop in satisfaction that is most likely explained by a lack of call center advisors speaking Bariba and Goun languages at that time. That year also saw a large turnover in call center staff, which likely caused some disruptions to service quality.

Although the adaptations to the Ligne Verte project over the years have been small, they have had a large impact on the callers' satisfaction. These adaptations required a fairly minimal investment. For example, providing headsets to call center advisors to reduce background noise and improve the call quality has positively improved the user experience. Other changes were more significant, including staff changes to expand the languages spoken offered by the service, allowing access for a greater number of potential callers who either live in more rural settings, are part of lower socioeconomic groups, or are less comfortable communicating in French.

Although the adaptations to the Ligne Verte program over the years have been small, they have had a large impact on the callers' satisfaction.

Some surprising findings have emerged from the annual evaluations. The first is that the majority of respondents to the evaluation were male, despite the service's focus on reproductive health and geared toward women. Selection of potential respondents for the annual evaluations is by simple random sampling and, therefore, is likely to broadly reflect the caller demographics. This suggests that the majority of Ligne Verte callers may also be male. Mobile phone ownership in Benin is skewed toward men (79.5% of men vs. 51.1% of women estimated to own a mobile phone),^8^ which may in part explain this result, but further research into barriers to accessing the helpline for women may be useful.

There are several recommendations that can be derived from the Ligne Verte improvement process. First, it is important to listen to clients' challenges and concerns. The Ligne Verte project has demonstrated that integrating end-user perspectives, especially from diverse ethnic, religious, linguistic, or socioeconomic backgrounds, can potentially add valuable insights to improve the reach and cost-effectiveness of the health program.^9^ To ensure that ideas from clients can be implemented, it is necessary to consider the budget for potential program adaptations ahead of deploying evaluations where possible and that recommendations for change come from open discussions between evaluators and implementers. Listening to clients may also provide opportunities for strengthening health system responsiveness beyond the helpline itself. As a study in Cote d'Ivoire demonstrated, helpline workers reporting COVID-19 rumors in real time provided rich insight and actionable insights into misinformation circulating in the wider population.^10^

Second, supporting staff morale should be prioritized for projects that have outcomes that depend on the provider's attitudes. Caller satisfaction from Ligne Verte was likely directly connected to the call center advisor's behavior. As demonstrated by other studies,^11^^,^^12^ the results from the Ligne Verte evaluations confirmed the need to invest in the well-being of service providers to maintain high-quality service provision.

Finally, as other health program evaluations have shown, instituting transparent and continuous feedback loops between all personnel involved in the project can potentially improve program effectiveness.^13^^–^^15^ As observed in the annual mini-workshops, convening front-end call center advisors with back-end health researchers and program managers created a working environment that fostered trust and collaboration among colleagues and permitted actionable recommendations to be developed.

### Limitations

The Ligne Verte annual satisfaction study is a telephone survey, meaning that interviewers cannot observe nonverbal communication from the respondents. Callers were eligible for inclusion if they had called Ligne Verte in the 3 months before data collection. For those who were at the earlier end of this time period, it is likely that recall biases may have affected their responses. In addition, although our measures of satisfaction have been consistent over time, it is not possible to account for changes in caller expectations, which likely conditioned their experiences. Future research might consider measuring client expectations as well as experience to better understand the relationship between them. The helpline did not collect routine data on caller age or gender, so it was not possible to assess the degree to which the Ligne Verte evaluation sample was representative of the total caller population. Men are more likely to own a mobile phone, particularly in rural areas of Benin, but it was unclear if the higher number of men in the evaluation sample was a result of them calling Ligne Verte more frequently than women or for another reason. Finally, many years of Ligne Verte evaluations have produced large volumes of data, but because of the limitations of space, we have only been able to present a small selection here. Nevertheless, we have aimed to give a representative overview of the insights gathered.

## CONCLUSION

Taking a consumer-centered approach, listening to user feedback, and making evidence-based recommendations to improve Ligne Verte have led to an overall increase in caller satisfaction. A success of this program's improvement process was the open and collaborative channels of communication between call center advisors, program management, and the research team. This approach allowed the team to develop a clearer understanding of the program's needs and weaknesses and thus make more realistic, affordable, actionable, and evidence-based recommendations to improve Ligne Verte. To create and maintain high client satisfaction, those designing and evaluating similar programs to Ligne Verte should prioritize collecting repeated data at regular intervals to assess levels of and changes in end-user and staff perspectives. Although not yet evident in end-user feedback, as smartphone ownership increases in Benin, we might expect increasing demands for a wider range of touchpoints (including chatbots and short message service). Ligne Verte evaluations will allow the project to monitor any changes in demand for other communication channels in the future.

## Supplementary Material

22-00296-Bouanchaud-Supplement.pdf

## Notes

**Peer Reviewed**

**First published online:** December 14, 2023.
