# Supplementary material for: Lessons Learned in Improving the Quality of a Free Reproductive Health Hotline in Benin
Source: Glob Health Sci Pract. 2023 Dec 22;11(6):e2200296. doi: 10.9745/GHSP-D-22-00296 (PMC10749658; doi:10.9745/GHSP-D-22-00296)
Supplement: 22-00296-Bouanchaud-Supplement.pdf [file 22-00296-Bouanchaud-Supplement.pdf]

Supplement to: Zinsou C, Guedegbe G, Dossou L, et al. Lessons learned in improving the quality of a free reproductive health hotline in Benin. *Glob Health Sci Pract*. 2023;11(6):e2200296. https://doi.org/10.9745/GHSP-D-22-00296

abms

membre du réseau psi

CONFIDENTIEL

GRILLE D'EVALUATION DE LA QUALITE DES SERVICES DE LA LIGNE VERTE

Commune_________________________________	***Nom de l'interviewer***_________________________
Date :	***Numéro d'identification /___/___/___/___/***

Bonjour. Je m'appelle____________________. Je travaille ponctuellement pour l'ABMS.
J'aimerais savoir si vous connaissez la ligne verte 7344 de l'ABMS : Oui /__/ Non /__/
Si oui, l'avez-vous appelée une fois ? Oui /__/ Non /__/ ***Si non, remerciez la personne et passez au suivant.***
Si oui*, nous sommes en train de faire une petite étude à propos de la satisfaction des usagers de la ligne verte et j'aimerais vous entretenir pendant 10 à 15 minutes si vous êtes disponibles. Si non, je pourrais vous rappeler après quand vous serez plus disponible.*

0.0	**Avez-vous plus de 18 ans ?**	***Oui 1 Non 2*** **Si non, aller à la fin du questionnaire**			
0.1	**0. Motif DE L'APPEL /___/**	**1- PF**	**2- VIH**	**3-Paludisme**	**4- Violence**
		**5- Conseils SSR**	**6- Déclaration Naissance**	**7- Mutuelle Santé**	**8- Autres**
	**1- ACCUEIL**	**Très satisfaisant**	**Satisfaisant**	**Pas satisfaisant**	**Pas du tout satisfaisant**
1-1	Avez-vous été bien accueilli par l'assistante ligne verte ? (Forme standard d'accueil. Ex: ABMS/PSI bonjour)	4	3	2	1
1-2	Avez-vous été invité à vous exprimer ? (ex: que puis- je pour vous Mr ou Mme?)	4	3	2	1

2- ECOUTE ACTIVE

2-1	L'assistante ligne verte vous a-t-elle laissé le temps de vous exprimer?	4	3	2	1
2-2	Avez-vous eu l'impression que l'assistante ligne verte vous a bien écouté et a bien compris votre préoccupation ?	4	3	2	1
	**3- SATISFACTION**				
3-1	L'assistante ligne verte vous a-t-elle fourni les réponses ou explications satisfaisantes à votre préoccupation?	4	3	2	1
3-2	L'assistante ligne verte s'est-elle assuré que vous avez été satisfait?	4	3	2	1
3-3	L'assistante ligne verte vous a-t-elle laissé vous exprimer dans la langue de votre choix ?	Oui 1	Non 2		
	**4- PRISE DE CONGES**				
4-1	L'assistante ligne verte s'est-elle assurée que vous avez exprimé toutes vos préoccupations?	4	3	2	1
4-2	L'assistante ligne verte a-t-elle reformulé votre préoccupation pour être sûre de l'avoir bien saisie (comprise)?	4	3	2	1
4-3	A-t-elle (il) pris congé de vous convenablement avant de raccrocher en disant par exemple : *Merci de nous avoir appelé, n'hésitez pas à nous rappeler en cas de besoin etc... ?*	4	3	2	1

5- APPRECIATION GENERALE

5-1	D'une façon générale, comment était le débit de la voix pendant l'entretien : Très rapide, rapide ou acceptable ?	Très rapide 1	Rapide 2	Acceptable 3
5-2	Le ton de la voix : était-il très bien, normal ou arrogant ?	Très bien 1	Normal 2	Arrogant 3
5-3	Les expressions utilisées étaient-elles très convenables, convenables ou peu convenables ?	Très convenable	Convenable	Peu convenable

Supplement to: Zinsou C, Guedegbe G, Dossou L, et al. Lessons learned in improving the quality of a free reproductive health hotline in Benin. *Glob Health Sci Pract*. 2023;11(6):e2200296. https://doi.org/10.9745/GHSP-D-22-00296

			1	2	3
5-4	Avez-vous été globalement satisfait de votre dernier appel 7344 ?		Oui 1	Non 2	
	**6. CARACTERISTIQUES SOCIODEMOGRAPHIQUES**				
6-1	Puis-je connaître votre profession ?	Elève<br>Etudiant (e)<br>Artisan (e)<br>Ménagère<br>Apprenti (e)<br>Commerçant (e)<br>Enseignant<br>Salarié (public/privé)<br>Agriculteur/pêcheur<br>Profession libérale<br>Autre___________________________<br>(préciser)	1<br>2<br>3<br>4<br>5<br>6<br>7<br>8<br>9<br>10<br>11		
6.2	Quel âge avez-vous ?	Âge en années révolues			
6-3	Sexe de l'enquêté (e)	Masculin<br>Féminin	1<br>2		

Qu'avez-vous encore à dire sur la ligne verte ?

____

____

____

____

____

Merci infiniment pour votre contribution à cette enquête
